# Practical Observations on Various Forms of Dyspepsia

**Published:** 1846-05

**Authors:** Robert Dick


### RECORD OF MEDICAL SCIENCE.

*Practical observations on various forms of Dyspepsia.* By Robert Dick, M. D.—Having thus considered the subject of primary and secondary assimilation, and its derangements, we shall now devote a few words to the treatment of indigestion in its complicated forms, namely, when it has given rise to derangements in distant organs. It may simplify this part of the subject, to state, at the outset, the general principles which, in these circumstances, must guide our measures. It is clear that the best way of curing the secondary, is by removing the primary affection: that, consequently, when directing our efforts to dissipate the dyspeptic derangements, we are in reality, though indirectly, acting against the lesions secondarily induced by it elsewhere. Accordingly, in all cases in which the secondary affection is of urgency and importance, measures directed to the original disease must ever constitute a part, and a considerable one, of the treatment. But secondary affections are sometimes so serious, so pressing, and involving such important organs, that to make their relief dependent altogether on the issue of measures directed to the original disease, would be to hazard imminently the life of the patient. For it seems certain, that chronic dyspeptic derangement may occasionally give rise, or dispose to, disease more urgent than itself. When this happens, *two series* of treatment must often be simultaneously pursued; the treatment of the secondary affection being grafted on that of the primary. This, I say, must often be done, it being not *always* necessary. When the secondary affection is trifling, transient, purely functional, it may not be necessary to hurry to measures distinct from those addressed to the original disease; but when the heart, lungs, brain, or spinal cord, betrays marked and persistent derangement, separate measures must be adopted.

In such circumstances we are sometimes under the necessity, on account of the secondary affection, of acting on the digestive organs in a way which we would avoid doing had we only the primary affection, or simple indigestion, to contend with. Thus, for a congestive headache, or for suspicious thoracic symptoms, we are occasionally compelled to administer powerful and debilitating purgatives. This is, however, a necessity to be avoided, if possible, and one, oftener than we might suppose, possible to be avoided: since the secondary affections are, in general, not so rapid as when occurring, primarily, in undebilitated constitutions. Chronic indigestion seems to give to morbid action the same indolent and feeble character which it induces in organs still comparatively healthy.

If the treatment of the primary affection, previous to the incidents of the secondary one, has been judicious, it is obvious that no alteration is required as regards that; all which is necessary is to superinduce the additional means required by the latter, and to redouble our attention. One circumstance demands notice. No advantage is derived to the secondary affection from the continued use of any medicine or plan of treatment, which aggravates the primary derangement. This is too obvious to require illustration. Digitalis, elaterium, colchicum, nux vomica, squills, though useful in anasarca and ascites, in gout, in paralysis, and pulmonary disease respectively, provided these organs are so prepared, or these medicines so combined, that no gastric excitement follows their employment, will, without these precautions, severally do harm: will both derange the assimilating surfaces, and likewise augment the dropsy, the arthritic, the paralytic, the pulmonary affection, in consequence of the febrile excitement they will induce; by which cutaneous transpiration is interrupted, and other constitutional disturbances created. Cases illustrating the justice of these observations must frequently present themselves to practitioners. Hence, the treatment of dyspeptic disease, when involving secondary affections, is somewhat nice and complex, and affords a more than ordinary opportunity for the display of genius and invention on the part of the physician.

*Functional and Structural Disease of the Heart, in connection with Derangement of the Digestive Organs.*—I believe that the only mode in which stomach complaint *directly* affects the heart, is a mechanical one, to wit, by means of the distension which enormous meals of solids and liquids, soups, fish, flesh, beer, wine, soda water, coffee, tea, all at a sitting, and the almost necessary flatulence, may cause, and by which, more especially if sleep is soon after indulged in, the diaphragm may be pressed up, and the heart's action impeded, but particularly the aorta may be seriously oppressed by the superjacent loaded stomach; and hence, the current of blood being opposed, the right ventricle is compelled unduly to exert itself. In this way I believe chronic gluttony may induce hypertrophy of the ventricle, nay, even aneurism of the aorta. In fact, I have witnessed cases, the history of which I believe to have been no other than this. I have also sometimes suspected that the palpitations which occasionally accompany prolonged derangements of the stomach, liver and bowels, may be owing to a preternaturally impure, and perhaps even acrid character of the portal circulation, and the impinging of a current of such blood against the internal lining membrane of the cava inferior and left auricle. But this idea has, of course, the value of mere conjecture. The only other modes in which dyspeptic derangements can affect the heart, are extremely circuitous. The one is by the channel of nerves, through, as I have already remarked, the intercommunion of the various parts of the sympathetic; the splanchnic nerve being the medium of transmitting irritation to one part of the ganglionic circle, the cardiac nerves reflecting it on the heart; or the par vagum may be the channel. A third mode in which chronic digestive derangement may affect the heart, is by the production of disorders of primary assimilation, by which constitutional complaints, such as gout, are induced, in the course of which the membrano-cartilaginous tissues of the heart may be structurally affected. Rules cannot be laid down for the treatment of such cases. The practitioner must discover and apply them for himself, in respect of each particular case. The palpitations due to inordinate meals, and to the injudicious use of flatulent articles, have an obvious remedy.

*Pulmonary Affections caused by Dyspeptic Derangements.*—This may appear in one or all of three ways:

1. By the propagation of inflammatory irritation along tissues common to both organs. This propagation may take place up the œsophagus and down the trachea; more circuitously, at least more obscurely, along the hepatic veins, lower cava, right auricle and ventricle, pulmonary artery; for it is easy to conceive how the blood, passing directly from an inflamed and morbid liver, may perniciously affect an organ of a structure and function so peculiar as that of the lungs. Of course, in this case, all attention must be given to quiet the digestive mucous surfaces.

2. Chronic digestive derangements may induce pulmonary disease, by causing a general and chronic febrile excitement, by which, more especially, if there is predisposition to chest affections, sudden and formidable disease of the lungs may be occasioned. It is a just remark of Andral, that it is necessary to watch, with the utmost jealousy, the slightest colds and pectoral symptoms of dyspeptics, since it is wonderful how insidiously the secondary pulmonary affection may grow into alarming independent disease.

A third and equally indirect mode in which protracted derangement of the assimilative organs, and of assimilation, may induce pulmonary disease, is one we have already referred to, in which, to requote Dr. Prout's expressions, "the imperfectly assimilated chyle, in passing through the lacteal system, either does not undergo the necessary changes by which chyle is converted into blood; or is malconverted into the comparatively insoluble pseudo-albuminous matter of struma, which, in passing through the lungs, lays the foundation, perhaps, at first mechanically, of tuberculous deposition and future accretion."* (Stomach and Renal Diseases, p. 490.) This is a cri-

* Pulmonary tubercle is probably various in its mode of formation as in its seat; being first formed now in the areolar tissue, now in the cells. As Dr. Prout seems to state, the granular and apparently nucleolar formation, is tical complication, requiring great judgment and promptitude on the part of the practitioner.

*Affections of the Brain and Spinal Cord, induced by Derangements of the Digestive Organs.*—The nerves of organic life may exhaust or irritate the spinal cord all along its tract, and through it the brain. The par vagum may more directly still produce a like effect.

Some recent writers on derangements of the digestive organs, and on the tendency of these to produce secondary affections, have too exclusively imputed the disorder of the brain, consequent on stomach complaints, to hyperæmia. Andral, in various parts of his works, makes observations corrective of this. "You will observe," he says, "sometimes the same symptoms in two individuals, the one of whom has his brain congested, the other anæmic." A recent practical writer observes: "it must be borne in mind, that the process of digestion is one of hyperæmia, or of active congestion of the mucous membrane of the stomach. This active congestion is repeated or reproduced in the brain in two ways, by the direct action of one organ on the other, or through the intervention of the heart. This state of stomach produces corresponding congestion of the brain," &c. This writer always assumes that what he calls active congestion is a natural state of the healthy stomach, and equally that "a corresponding congestion of the brain," healthy or not, necessarily takes place. I question both affirmations, particularly the latter. And I apprehend that many of the phenomena supposed unquestionably due to cerebral congestion consequent on digestive activity of the stomach are to be imputed to other causes. This may be illustrated by a somewhat analogous case. A patient of Dr. Sweatman's had kept the horizontal posture ten years. If she sat up for a short time she fainted: yet, during this period she had borne children, and appeared in health. Dr. S., on examining the patient, found immense varices in the veins of her legs, into which, when she stood up, an immense quantity of blood gravitated, which was the cause of the deliquium. She was cured by her legs being bandaged. Now I apprehend that the drowsiness, the temporary hebetude, which are stated by the gentleman just referred to as infallible proofs of an hyperæmic state of the brain, consequent on a corresponding state of the stomach, during the digestive process, are, I shall not say always, but frequently due to a condition of the nervous power, and of its circulating fluids, analogous to what took place in the case of this lady. It is to the concourse of blood towards the stomach, liver, and intestines, and the consequent subduction of it from the cerebrum, but perhaps more particularly to a subduction of nervous power and activity, analogous often posterior to the depotism of the tubercular matter. "Die tuberculöse infiltration besteht in der verwandlung einer unbestimmt begranzten grösseren parthie des lungasgewebes in tuberculöse kässige masse: die infiltration ist nicht erst aus dem zutammen-fliessen isolirter tuberkörner (durch aggregration) anstanden die granulationen entwickeln sich oft erst spöter aus der infiltration." Canstall's Handbuch der medicinischen Klinik, page 812 Consult also Rokitanski, Hasse, &c.

to that of the blood, from the brain to the stomach, there to supply the additional amount of nervous power required during digestion, that drowsiness and other cerebral symptoms noticed at this period are chiefly due. Chronic dyspeptics do not usually superabound with blood; and why or how the brain, unless disposed to disease, should become hyperæmic simultaneously with the digesting stomach, is not easy to see; while it is, on the other hand, obvious, that it would require considerable general plethora to admit of these organs being simultaneously hyperæmic. It is, moreover, to be kept in mind, that chronic dyspeptics are usually, from bitter experience, extremely circumspect in diet; though, indeed, there are many exceptions to this. I am, therefore, disposed to explain the cerebral excitement or fulness, occasionally accompanying digestion or dyspeptic derangements, as caused through the medium of the sympathetic; the ultimate branches of which, netted on all the minute twigs of the carotids, and entering with these into the inmost recesses of the brain, may produce singular irregularities of the function and circulation of that organ. Congestion of the brain, even effusions in it, pains, throbbings, and lesion or anomalous action of the sensal organs, may each have the source now indicated.

In all such cases the liver must receive attention, and this point is of the more importance, as usually, when the liver acts inefficiently. the kidney also does so. A free state of the digestive surfaces will in most cases go far to relieve the sympathetic affections of the cerebro-spinal organs, which depend on moderate plethora and irritable congestion. Ipecacuanha joined to mild purgatives, and administered for some time, is conspicuously advantageous here. If great irritability of the gastric organs is present, and we have reason to conjecture that the ganglionic nerves participate in this state, as proved by cardiac palpitations, oppressed or accelerated respiration, headache, &c., then hyoscyamous, camphor, ipecacuan, must be freely given; the bowels being kept in lax but not draining action. If the bowels and kidneys, by the means directed for the purpose, act with tolerable freedom, we need not insist on a sensibly perspiring state of the skin. Yet a somewhat moist state of it should be certainly secured, since on its being established spontaneously, or by medicine, some of the most formidable head affections and pains about the lumbar spine pass away, almost without any other means.

If there be cerebral but not general plethora—that is, if, though the head be hot, the cheeks red, the conjunctiva tumid, and a sensation within the cranium of fulness, distension, or throbbing be felt, there be yet a pulse calm and only moderately full, then leeching in the fossæ behind the ears, or cupping on the nape of the neck, followed by a blister, if it appear necessary, will be required. "The most effectual remedy," observes Dr. Philip, when speaking of sympathetic headache, "is bloodletting from the head or its immediate neighborhood." But if the plethora be general, and the gastric affection of an irritable hyperæmic kind, leeches must be applied to the epigastrium, and also to the anus, and saline purgatives be given. Dr.

Philip speaks of emetics, which I cannot recommend except very occasionally: to wit, when the headache is entirely due to some indigestible aliment lodged in the stomach, and within an emetic's reach; for if it be passed into the duododenum purgatives are better, since ingesta can be recovered from the duodenum only by unpleasantly violent anti-peristaltic action. Yet the principle on which Dr. P. proposes to administer both emetics and cathartics is strictly sound. "Emetics and cathartics frequently relieve headache, because they not only tend to remove the cause of headache, but occasion—the former immediate, the latter eventual—depletion of the vessels of the head."

In anæmic headache the course is different. This headache may not be directly due to gastric derangement, yet it is to be cured in great part by means addressed to the digestive organs. Thus, for example, child-bed flooding, or any other resembling cause, may produce intense headache, and intense pain and debility of stomach, simultaneously; but in such a case we must neither impute the headache to the gastric affection, nor the gastric affection to the headache. They are, in such cases (and these cases are not rare,) synchronous, or, if we may be allowed to coin a word possibly a little more exact, *iso-chronic* affections, dependent on a common cause: to wit, anæmia of the cerebro-spinal organs, and languor of the circulation. Yet the treatment of both must be chiefly through the stomach.

Let us now very briefly notice digestive derangements secondarily produced from affections of distant parts and organs. And first, as regards cutaneous derangements. The effects of an extensive scorch or scald of this surface will perhaps best illustrate its pathological relations with the digestive organs. In the event of such an accident, the nervous centres are powerfully excited and exhausted; and at the same time, it often happens that one or other of the mucuous canals, alimentary or urinary, is inflamed. Our business, at present, is with the former. This, then, especially if there have been predisposition to disease, is not unusually precipitated by the accident referred to into acute inflammation; it rarely fails, at least, to manifest a considerable degree of secondary disturbance. The cutaneous and mucous surfaces are, as we have before observed, antagonistic; that is, an active secreting mucous membrane causes a less active cutaneous, and *vice versa.* Now, as was also formerly remarked, and as, in fact, follows from the very terms of the proposition now stated, membranes that antagonize also supplement each other; that is, if the cutaneous membrane, by sordes, by eruptive or other disease, by scald, scorch, &c., is rendered incapable of its function, the mucous membrane is rendered more active (in common, it may be, with the urinary,) and may thus have a vicarious duty suddenly thrown upon it, which, if already morbidly disposed, it may not be unable to undertake without excitement verging on inflammation. On this principle, then, we explain dyspeptic derangements consequent on or accompanying small pox, measles, scarlatina, &c.

Disease of the heart produces secondary indigestion, or hepatic hyperæmia, principally by causing congestion of the gastric mucous surfaces, and of the portal system.—*Lond. Med. Gaz.*

